# Interrelationship between 2019-nCov receptor DPP4 and diabetes mellitus targets based on protein interaction network

**DOI:** 10.1038/s41598-021-03912-6

**Published:** 2022-01-07

**Authors:** Qian Gao, Wenjun Zhang, Tingting Li, Guojun Yang, Wei Zhu, Naijun Chen, Huawei Jin

**Affiliations:** Affiliated Hospital of Shaoxing University of Endocrine and Metabolism Department, Zhejiang, China

**Keywords:** Drug development, Epidemiology, Outcomes research

## Abstract

Patients with diabetes are more likely to be infected with Coronavirus disease 2019 (COVID-19), and the risk of death is significantly higher than ordinary patients. Dipeptidyl peptidase-4 (DPP4) is one of the functional receptor of human coronavirus. Exploring the relationship between diabetes mellitus targets and DPP4 is particularly important for the management of patients with diabetes and COVID-19. We intend to study the protein interaction through the protein interaction network in order to find a new clue for the management of patients with diabetes with COVID-19. Diabetes mellitus targets were obtained from GeneCards database. Targets with a relevance score exceeding 20 were included, and DPP4 protein was added manually. The initial protein interaction network was obtained through String. The targets directly related to DPP4 were selected as the final analysis targets. Importing them into String again to obtain the protein interaction network. Module identification, gene ontology (GO) analysis and Kyoto encyclopedia of genes and genomes (KEGG) pathway analysis were carried out respectively. The impact of DPP4 on the whole network was analyzed by scoring the module where it located. 43 DPP4-related proteins were finally selected from the diabetes mellitus targets and three functional modules were found by the cluster analysis. Module 1 was involved in insulin secretion and glucagon signaling pathway, module 2 and module 3 were involved in signaling receptor binding. The scoring results showed that LEP and apoB in module 1 were the highest, and the scores of INS, IL6 and ALB of cross module associated proteins of module 1 were the highest. DPP4 is widely associated with key proteins in diabetes mellitus. COVID-19 may affect DPP4 in patients with diabetes mellitus, leading to high mortality of diabetes mellitus combined with COVID-19. DPP4 inhibitors and IL-6 antagonists can be considered to reduce the effect of COVID-19 infection on patients with diabetes.

## Introduction

Due to the high prevalence and long incubation periods often without symptoms, the severe acute respiratory syndrome coronavirus-2 (SARS-CoV-2) has infected hundreds of millions of people around the world, causing the coronavirus disease 2019 (COVID-19) pandemic^1^. Recent studies have shown that dipeptidyl peptidase-4(DPP4) is one of the functional receptor of human coronavirus^2–5^. SARS-CoV-2 Virus S protein can infect the body by acting on this receptor of bronchiolar epithelial cells^6^. DPP4 also known as CD26, is a serine exopeptidase, a multifunctional type-II transmembrane glycoprotein that presents in a dimeric form on the cell surface. DPP4 is multifunctional, highly conserved among mammals. It regulates the activities of peptide hormones, neuropeptides, cytokines and growth factors, but also act as a surface antigen to cooperate with other molecules or proteins to mediate the interaction between cells and matrix, cells and cells, and play various regulatory roles in immune activation, inflammatory response and tumorigenesis^7^.

DPP4 inhibitors are a kind of hypoglycemic drugs which have been widely used in patients with diabetes. DPP4 can decompose glucagon like peptide-1 (GLP-1), which can stimulate insulin secretion. DPP4 inhibitors can effectively antagonize this effect and then control blood glucose, especially postprandial blood glucose, and improve glucose tolerance, insulin resistance and other symptoms of patients by inhibiting the degradation of GLP-1^8^. Patients with diabetes are more likely to be infected with COVID-19, and the risk of death is significantly higher than ordinary patients^9,10^. Current studies have shown that DPP4 inhibitors can be considered as the preferred hypoglycemic regimen in the treatment of patients with diabetes with COVID-19 infection^11^. Exploring the relationship between DPP4 and diabetes targets is particularly important for the management of patients with diabetes and COVID-19.In order to strengthen the management of patients with diabetes with COVID-19, we intend to study the protein interaction through the protein interaction network, and evaluate the degree of protein–protein correlation through the correlation score. Based on the String database, this paper analyzes the relationship between DPP4 and diabetic protein targets, in order to find a new clue for the management of patients with diabetes with COVID-19.

## Materials and methods

### Get diabetes mellitus targets information

Taking ‘diabetes mellitus’ as the keyword, diabetes mellitus-related targets were searched in GeneCards database (https://www.genecards.org/). The target with a relevance score exceeding 20 was selected as the research target.

### Screening of protein targets directly associated with DPP4

The diabetes mellitus related target (obtained by step 2.1) and DPP4 were imported into STRING database (https://string-db.org/) ^12^ in the form of symbol to obtain the protein–protein interaction network map. And then the targets directly related to DPP4 were screened for further research.

### Construction of DPP4 related protein network

DPP4 and 43 related proteins were re-imported into STRING to obtain the protein–protein interaction network for subsequent analysis.

### Identification and analysis of DPP4 related protein network modules

In the STRING website, K-means algorithm was used to cluster the DPP4 and 43 related proteins.

### Gene ontology (GO) and Kyoto encyclopedia of genes and genomes (KEGG) pathway analysis of each module

The enrichment analysis of GO function and KEGG pathway analysis was carried out through STRING to predict its action mechanism and construct the network diagram.

### Evaluation of the impact of DPP4 on the whole protein network

In order to further evaluate the impact of DPP4 on the whole network and the possible pathway, the module 1 where DPP4 is located is used as the internal network, the cross module association of internal network is screened out. The statistical description and mapping are carried out. The data are filtered with 0.6 (determined by mean and median) as the standard, the qualified cross module associated proteins and associated scores were listed, and the proteins included in module 1 and the cross module associated protein scores of module 1 were summed as the scores.

## Results

### Screening results of DPP4 associated proteins

A total of 1031 diabetes mellitus targets and 43 targets directly related to DPP4 were obtained by screening (Table 1).

**Table 1:** DPP4 related protein.

Symbol	Protein name	Score
ACE	Angiotensin-converting enzyme	0.709
ADIPOQ	Adiponectin	0.572
AKT1	RAC-alpha serine/threonine-protein kinase	0.52
ALB	Serum albumin	0.671
APOB	Apolipoprotein B-100	0.438
APOE	Apolipoprotein E	0.422
CAV1	Caveolin-2	0.692
CCL2	C–C motif chemokine 2	0.448
CCR5	C–C chemokine receptor type 5	0.4
CPE	Carboxypeptidase E	0.438
CRP	C-reactive protein	0.536
CTLA4	Cytotoxic T-lymphocyte protein 4	0.44
CXCL10	C-X-C motif chemokine 10	0.581
FN1	Fibronectin type III domain containing	0.773
GCG	Glucagon	0.994
GCGR	Glucagon receptor	0.573
GCK	Glucokinase	0.461
GGT1	Glutathione hydrolase 1 proenzyme	0.411
GHRL	Appetite-regulating hormone	0.559
GLP1R	Glucagon-like peptide 1 receptor	0.899
GPT	Alanine aminotransferase 1	0.508
HNF1A	Hepatocyte nuclear factor 1-alpha	0.538
IAPP	Islet amyloid polypeptide	0.672
ICAM1	Intercellular adhesion molecule 1	0.441
IL10	Interleukin-10	0.41
IL6	Interleukin-6	0.514
INS	Insulin	0.942
INS-IGF2	Insulin, isoform 2	0.535
LEP	Leptin	0.593
MMP2	72 kDa type IV collagenase	0.404
MMP9	Matrix metalloproteinase-9	0.405
NOS3	Nitric oxide synthase, endothelial	0.658
NPY	Pro-neuropeptide Y	0.735
PPARG	Peroxisome proliferator-activated receptor gamma	0.51
REN	Renin	0.499
SERPINE1	Plasminogen activator inhibitor 1	0.454
SLC2A2	Solute carrier family 2, facilitated glucose transporter member 2	0.462
SLC2A4	Solute carrier family 2, facilitated glucose transporter member 4	0.461
SLC5A2	Sodium/glucose cotransporter 2	0.892
SST	Somatostatin	0.472
TNF	Tumor necrosis factor	0.473
VCAM1	Vascular cell adhesion protein 1	0.416
VEGFA	Vascular endothelial growth factor A	0.643

### Protein interaction network results

**Figure 1:**
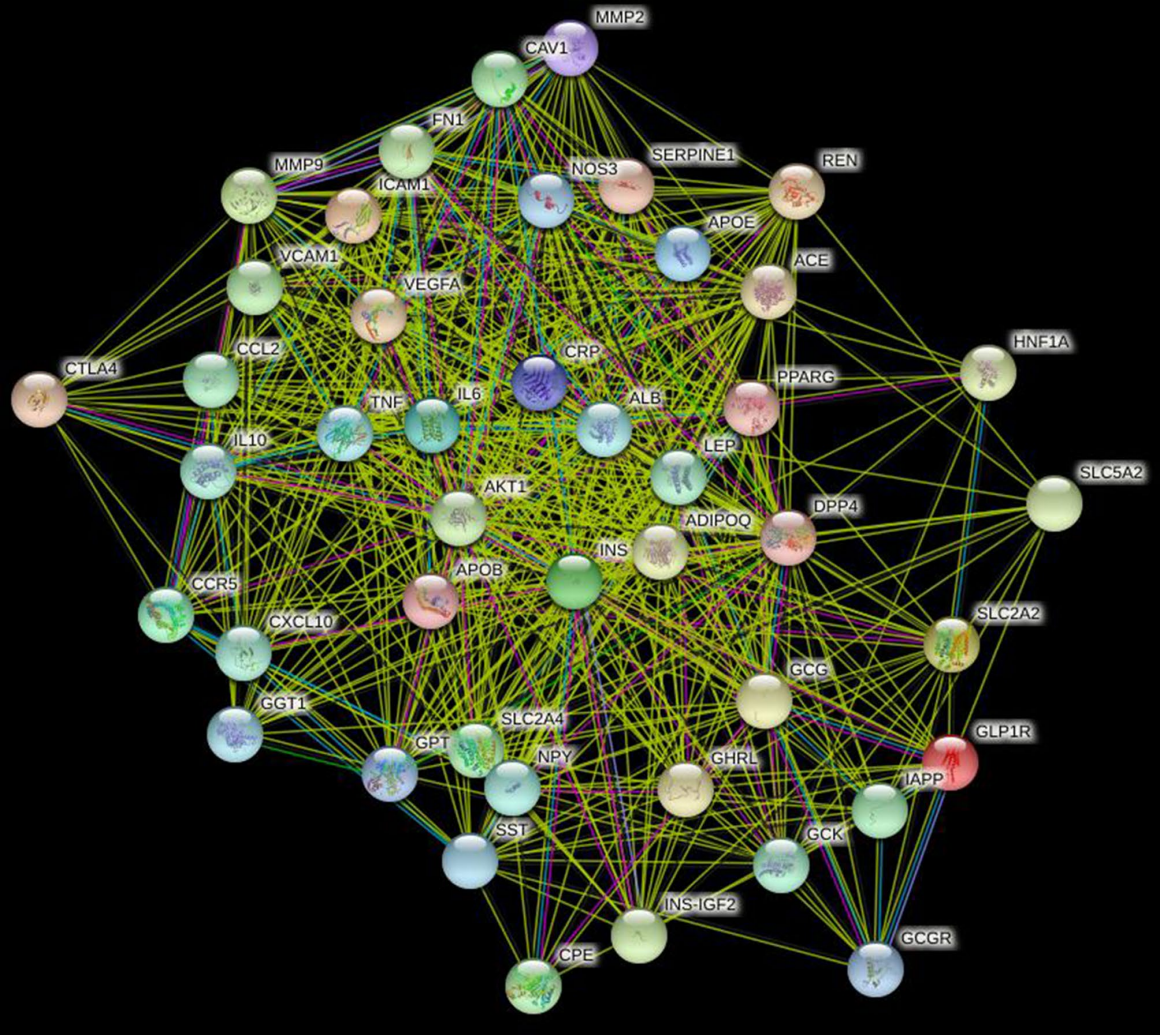
Interaction networ of DPP4 related protein.

The network consists of 44 nodes and 570 edges. The average value of nodes is 25.9 (Fig. 1).

### Cluster analysis results of protein interaction network

**Figure 2:**
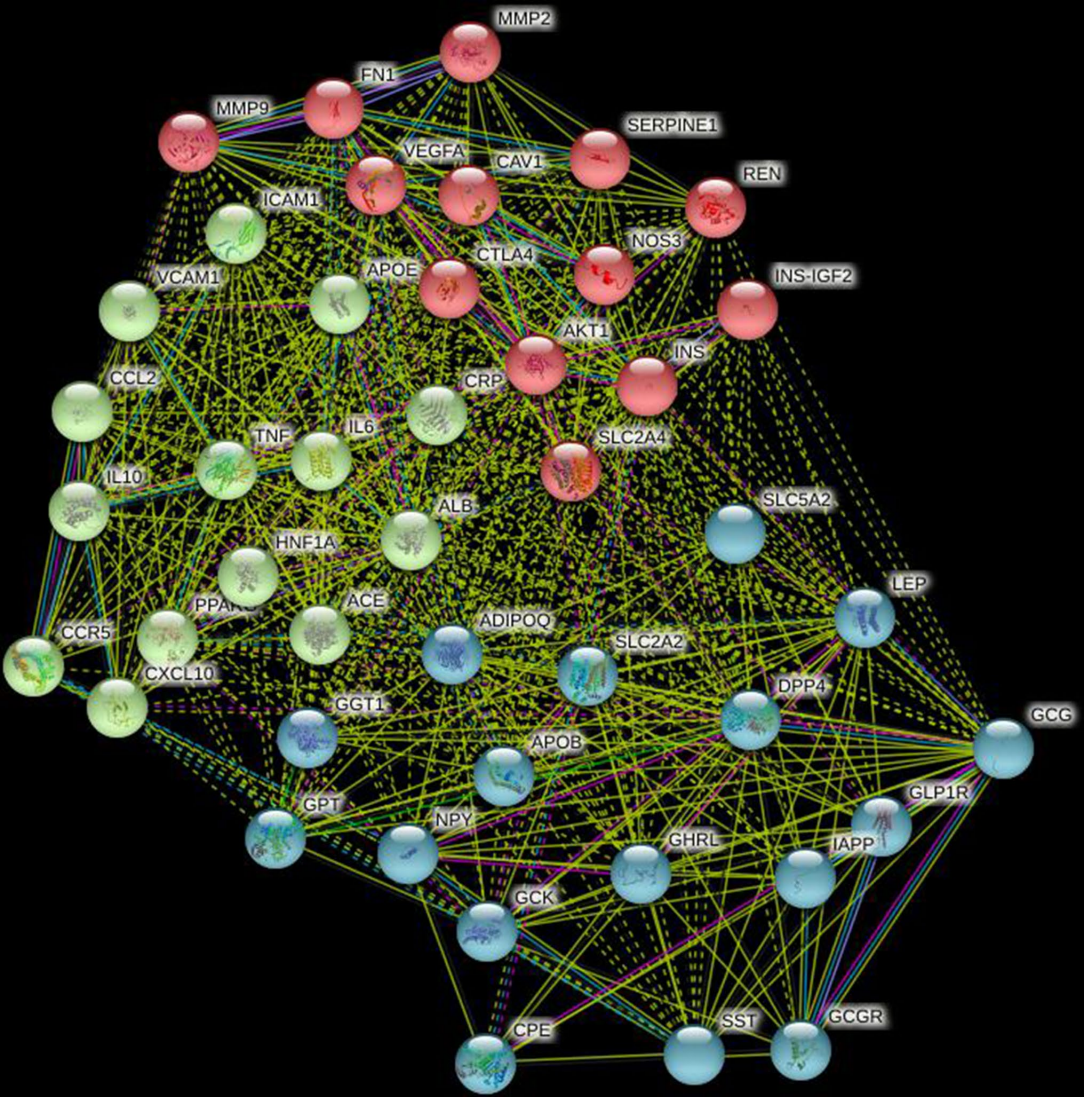
DPP4 related protein module recognition.

Three functional modules were obtained by cluster analysis. Module 1 includes 17 nodes (SLC5A2, SLC2A2, LEP, GCG, DPP4, ADIPOQ, APOB, GGT1, GPT, NPY, GCK, CPE, SST, GCGR, IAPP, GLP1R, GHRL); module 2 includes 14 nodes (ICAM1, VCAM1, APOE, CCL2, TNF, CRP, IL6, ALB, ACE, HNF1A, CCR5, CXCL10, IL10, PPAARG); module 3 includes 13 nodes (MMP2, MMP9, FN1, VEGFA, CAV1, CTLA4, NOS3, AKT1, INS, SLC2A4, REN, SERPINE1, INS-IGF2) (Fig. 2).

### GO analysis and KEGG analysis

Through GO analysis and KEGG analysis, it was found that the target of module 1 was mainly enriched in insulin secretion and glucagon signal transduction pathway; the target of module 2 and module 3 was mainly enriched in signaling receptor binding.

### Module 1 cross module correlation statistics results

Module 1 contains 190 cross module associations, accounting for 33% of the total number of network associations (570). The average score of association is 0.649, and the median is 0.628 (Table 2).

**Table 2:** GO and KEGG analysis of different modules.

Module	Protein	GO and KEGG analysis	*P*
Module 1	SLC5A2, SLC2A2, LEP, GCG, DPP4, ADIPOQ, APOB, GGT1, GPT, NPY, GCK, CPE, SST, GCGR, IAPP, GLP1R, GHRL	GO: hormone activity KEGG: Maturity onset diabetes of the young; Insulin secretion; Glucagon signaling pathway	1.29e−07 3.55e−05 3.55e−05 3.55e−05
Module 2	ICAM1, VCAM1, APOE, CCL2, TNF, CRP, IL6, ALB, ACE, HNF1A, CCR5, CXCL10, IL10, CCL2, PPAARG	GO: signaling receptor binding KEGG: African trypanosomiasis; TNF signaling pathway	2.63e−05 9.61e−08 9.61e−08
Module 3	MMP2, MMP9, FN1, VEGFA, CAV1, CTLA4, NOS3, AKT1, INS, SLC2A4, REN, SERPINE1, INS-IGF2	GO: signaling receptor binding KEGG: Fluid shear stress and atherosclerosis	0.0030 2.14e−08

### Module 1 cross module correlation impact assessment results

In module 1, LEP and apoB have the highest scores, which indicate that the above proteins may have cross module effects. The scores of INS, IL6 and ALB of cross module associated proteins of module 1 were the highest, which indicates that the effects of module 1 on other modules are more likely to be achieved through interaction with the above proteins (Table 3).

**Table 3:** Module 1 cross module associated protein and relevance score.

APOB		GGT1		GPT		NPY		CPE	
NOS3	0.707	IL6	0.616	IL10	0.66	IL6	0.709	INS	0.97
VCAM1	0.74	CRP	0.669	TNF	0.707	INS	0.889		
IL10	0.751	INS	0.67	IL6	0.733	CXCL10	0.908		
MMP9	0.789	ALB	0.805	INS	0.839	CCR5	0.919		
TNF	0.793			CRP	0.896				
INS	0.848			ALB	0.924				
IL6	0.969								

SST		GCGR		GHRL		GCG		LEP	
ALB	0.621	INS	0.694	INS-IGF2	0.607	INS-IGF2	0.668	REN	0.662
AKT1	0.68			NOS3	0.628	SERPINE1	0.672	CAV1	0.682
CXCL10	0.908			IL6	0.63	ALB	0.704	INS-IGF2	0.7
INS	0.915			ALB	0.675	AKT1	0.776	MMP9	0.707
CCR5	0.922			AKT1	0.71	SLC2A4	0.793	VCAM1	0.722
				INS	0.944	INS	0.986	MMP2	0.724
								CCL2	0.732
								ICAM1	0.762
								NOS3	0.762
								APOE	0.775
								IL10	0.797
								ALB	0.803
								AKT1	0.841
								SLC2A4	0.856
								VEGFA	0.867
								SERPINE1	0.879
								TNF	0.9
								IL6	0.943
								INS	0.976
								CRP	0.981

## Discussion

By sorting the correlation scores of DPP4 related diabetes targets, we found that GCG, INS and GLP1R had the highest correlation scores, which were more than 0.8, and GCG and INS were more than 0.9, which meant that DPP4 was most closely related to the above diabetes targets. By cluster analysis, three functional modules were found. Module 1 which contain DPP4 was mainly involved in insulin secretion and glucagon signal transduction pathway, while module 2 and module 3 were involved in signaling receptor binding. The binding of S protein of COVID-19 with DPP4 was the starting point of COVID-19. In order to evaluate the possible pathogenesis of the virus after entering the human body, DPP4 was taken as the starting point of the whole network for in-depth analysis. Because there were a lot of low values in the correlation scores of DPP4 cross module associated proteins, the mean and median were used as the standard to filter the data, and the sum of the correlation scores was used as the score. It was found that the scores of LEP and apoB were the highest in module 1. The scores of INS, IL6 and ALB of cross module associated proteins of module 1 were the highest. It is worth noting that DPP4 cross module correlation scores are less than 0.6, which do not been shown. Therefore, the abnormal changes of DPP4 may not play an effect by directly interacting with module associated proteins, but may be that module 1 magnifies its effect, and this effect is transmitted to module 2 and module 3 through the close relationship between module 1 and INS, IL6 and ALB, resulting in the disorder of glucose metabolism and inflammatory regulation.

Clinical trials show that patients with diabetes were more likely to be infected with SAR-COV-2, while the prognosis of patients with diabetes was worse and the risk of death was higher^9,10^. So, what was the mechanism of susceptibility to SAR-COV-2 in patients with diabetes? What was the mechanism of higher risk of death and worse prognosis in patients with diabetes? How to guide the medication of patients with diabetes infected with SAR-COV-2 in clinic? DPP4 as a type II transmembrane protein was also known to be cleaved from the cell membrane involving different metalloproteases in a cell-type-specific manner. Circulating, soluble DPP4 had been identified as a adipokine, which exerts both para- and endocrine effects^13^. Recently, studies found that sCD26 serum protein levels are reduced in diabetes. High serum sCD26 level could protect from viral infection by blocking the receptor from virus entry, whereas low sCD26 level may be associated with a higher risk of infection which may be one of the mechanisms of susceptibility to SARS-COV-2 in patients with diabetes^14^. The high mortality of patients with COVID-19 was closely related to the disorder of glucose metabolism and inflammation regulation^15^. The S protein of SARS-COV-2 can invade T, NK and other immune cells through binding receptor DPP4, and activate nuclear factor—κ B (NF—κ b) pathway, resulting in the secretion of a series of pro-inflammatory cytokines, including IL-6^16^. IL-6 can promote the differentiation of T helper cell 17 (Th17) and other lymphocyte changes. Circulating IL-6 and soluble IL-6 receptor complexes indirectly activate many types of cells, including endothelial cells, leading to the proliferation of a series of cytokines, leading to decreased blood pressure and acute respiratory distress syndrome (ARDS)^17^. IL-6 plays a key role in this cascade. It suggest the possibility of IL-6 antagonists (such as tocilizumab, sarilumab and siluximab) been used in severe COVID-19 disease. DPP4 also degraded GLP-1 and Gastric Inhibitory Polypeptide (GIP) and played an important role in glucose metabolism. Studies shown that GLP-1-based therapy can reduce the activation of immune cells, inhibit the release of pro-inflammatory cytokines, and reduce organ dysfunction and mortality^18^. DPP4 inhibitor increased the half-life of GLP-1 and therefore prolonged the half-life of insulin. Because of this, DPP4 inhibitor became a major target for the treatment of patients with diabetes. DPP4 inhibitors can also resist lung inflammation and reduce lung injury^19^. These studies suggested that DPP4 inhibitors may play an active role in the treatment of patients with diabetes with COVID-19. However, the issue remained controversial. Males believed that DPP4 inhibitors can inhibit the immune system and may increase the risk of infection^20^. However, there were also studies show that DPP4 inhibitors do not have a negative impact^21^. Previous retrospective studies found that DPP4 inhibitors have serious heterogeneity in the treatment effect of COVID-19^22–28^. However, these studies were not randomized controlled double-blind studies. At present, three randomized controlled trials (NCT 04341935, NCT 04371978 and NCT04365517) (Retrieved from: https://clinicaltrials.gov/) were ongoing to study the effect of DPP4 inhibitors on the prognosis of COVID-19 and the clinical results were expected to be obtained as soon as possible.

Leptin which was a hormone secreted by adipose tissue had the highest cross module effect in module 1, with a score of 16.071. When the body fat was reduced or in a low-energy state (such as starvation), leptin decreased significantly, thus stimulating the food seeking behavior and reducing its own energy consumption. On the contrary, when the body fat increased, leptin increased, which inhibited eating and accelerates metabolism^29^. Leptin was also associated with monocyte activation and severe illness in patients with COVID-19^30^. Overweight patients with COVID-19 tended to have higher leptin levels, which further activated monocytes, leading to amplification or imbalance of immune response, which may also be the mechanism of overweight patients more prone to serious diseases^31^. Cytokine release syndrome was an important cause of death in patients with COVID-19. However, the changes of cytokine profile and the underlying mechanism were still unknown. Using the cytokine array containing 174 cytokines related to inflammation found that the cytokine spectrum of severe patients with COVID-19 was significantly different from that of mild patients or healthy controls. Leptin, CXCL-10, IL-6, IL-10, IL-12, TNF and other cytokines, indicating that these inflammatory factors can predict the severity of COVID -19 disease^32^. The cluster analysis showed that leptin in module 1 and IL-6, IL-10, TNF in module 2 had a cross model effect, and the highest score of IL-6 is 6.163, which further supported the possibility of IL-6 antagonists in the treatment of severe COVID-19 disease.

In this paper, we screened out the diabetes targets and functional modules closely related to DPP4 through protein interaction network, and analyzed the influence of DPP4 module 1 on the whole protein network and the possible pathway. It was proposed that the influence of COVID-19 infection was amplified by DPP4 in patients with diabetes, and through its interaction with INS, leptin, IL-6 and other proteins. Increased glucose metabolism disorder and excessive inflammatory reaction lead to the high mortality in patients with diabetes with COVID-19. At present, the data of retrospective observational studies showed that the therapeutic effect of DPP4 inhibitors had serious heterogeneity, but the strength of the above studies was low. We look forward to further randomized controlled trials to verify our inference.

## Conclusions

DPP4 was widely associated with key proteins in diabetes mellitus. COVID-19 may affect DPP4 in patients with diabetes mellitus, leading to high mortality of diabetes mellitus combined with COVID-19. DPP4 inhibitors and IL-6 antagonists can be considered to reduce the effect of COVID-19 infection on patients with diabetes.
